# Unpleasant Smell: A Case Report of Trimethylaminuria (Fish Odour Syndrome) in a Child

**DOI:** 10.7759/cureus.79318

**Published:** 2025-02-19

**Authors:** Maria M Resende, Laura Leite-Almeida, Patricia Campos, Inês Sobreira, Paula Garcia

**Affiliations:** 1 Department of Pediatrics, Unidade Local de Saúde da Região de Aveiro, Aveiro, PRT; 2 Department of Pediatrics, Unidade Local de Saúde de São João, Porto, PRT; 3 Department of Pediatrics, Centro Hospitalar Universitário de Santo António, Unidade Local de Saúde de Santo António, Centro Materno-Infantil do Norte Albino Aroso, Porto, PRT

**Keywords:** fish odour syndrome, flavin-containing monooxygenase 3, metabolic disorders, trimethylamine, trimethylamine n-oxide, trimethylaminuria

## Abstract

We report a case of trimethylaminuria, also known as fish odour syndrome, in a child. This rare autosomal recessive metabolic disorder is caused by homozygous or compound heterozygous mutations in the FMO3 gene, which encodes the protein flavin-containing monooxygenase 3 (FMO3). The impaired function of this enzyme results in the accumulation of trimethylamine (TMA), a volatile, odouriferous compound excreted in the breath and bodily fluids that emits the characteristic odour of rotting fish.

A previously healthy three-year-old boy began exhibiting a rotting fish-like body odour at 10 months of age after consuming swordfish. Subsequent episodes occurred with the ingestion of other types of fish. A fish-free diet temporarily resolved the odour, but symptoms recurred when fish was reintroduced. The child’s growth and neurodevelopment were normal, and no abnormalities were detected during physical evaluation. Genetic testing revealed heterozygous variants in the FMO3 gene, including the intronic variant c.627+10C>G and the polymorphism c.472G>A (p.Glu158Lys), which, in combination, had the potential to cause moderate or transient symptoms. Dietary management with gradual fish reintroduction and hygiene measures were implemented. By 19 months of age, the child was consuming the recommended portions of fish without recurrence of the odour or any other symptoms.

This case illustrates a transient form of trimethylaminuria, likely resulting from a combination of identified genetic variants and the FMO3 enzymatic immaturity typical in early childhood. Awareness of this condition is essential for prompt diagnosis and effective management. Dietary and hygiene strategies can effectively alleviate symptoms and improve quality of life. This case also underscores the potential spectrum of trimethylaminuria phenotypes and the value of personalised management strategies.

## Introduction

Trimethylaminuria, or fish odour syndrome, is a rare autosomal recessive metabolic disorder characterised by a distinctive body odour resembling that of rotting fish. This unpleasant odour results from the excessive excretion of trimethylamine (TMA) in bodily secretions, including urine, sweat, and breath. Under normal metabolic conditions, dietary precursors such as trimethylamine-N-oxide (TMAO), lecithin, and choline are converted to TMA by gut bacteria. TMA is then transported to the liver, where the enzyme flavin-containing monooxygenase 3 (FMO3) oxidizes it to the odourless TMAO, which is subsequently excreted in the urine [1-7]. Under physiological conditions, 90% or more of the total TMA is typically excreted as TMAO [3,4].

Trimethylaminuria can be classified into two major forms. The primary form results from genetic alterations in the gene encoding the FMO3 enzyme, leading to a decrease in FMO3 function. The secondary form results from an overload of TMA or its precursors, such as a result of high dietary intake, liver disease, intestinal bacterial overgrowth (e.g., due to chronic kidney disease), or hormonal changes. In addition to these two forms, other minor transient forms are considered, one occurring in childhood and the other in women associated with menstruation [1,2,7].

Owing to the rarity of trimethylaminuria, its global incidence is not well established. Epidemiological studies have found that approximately 0.5%-1% of the British Caucasian population are carriers of a disease-causing FMO3 gene variant. Given that the pattern of inheritance is autosomal recessive, this corresponds to an estimated incidence of affected individuals as high as one in 40,000 [6,7]. This incidence is likely underestimated, and carrier rates may be higher in other populations [1,5,7].

Diagnosis of trimethylaminuria is primarily clinical, with a physical examination typically being unremarkable aside from the odour. Confirmation of diagnosis should include genetic testing and quantitative analysis of TMA and TMAO levels in the urine, when available [1-5,7]. We report a case of transient trimethylaminuria in a three-year-old boy.

This study was previously presented as a poster at the 9th Congress of the European Academy of Paediatric Societies in 2022.

## Case presentation

We present the case of a three-year-old Portuguese boy who was exclusively breastfed until six months of age, after which he began food diversification. Fish was introduced into his diet at seven months old, with no apparent complaints until he reached 10 months of age. At that point, after swordfish ingestion, his mother noticed an unusual and intense odour, resembling ‘rotting fish’, predominantly from his head and hands. Since then, similar episodes occurred with other types of fish. At first, his mother decided to try a fish-free diet for two months, during which no symptoms recurred. At 12 months of age, hake and subsequently forkbeard were introduced into the child’s diet with the reappearance of the fish-like odour.

The child’s growth and neurodevelopment were normal, and during physical evaluation, no malodour or other abnormalities were noted. However, the child had not consumed any fish prior to the examination. No similar symptoms were reported by the parents or any other family members, and there was no history of parental consanguinity. All laboratory tests, including biochemical markers for renal, thyroid, and hepatic function, were normal.

Trimethylaminuria was suspected, and the case was discussed and managed collaboratively with a metabolic paediatrician. A molecular study of the FMO3 gene was requested, which revealed heterozygous variants in the FMO3 gene, specifically the intronic variant c.627+10C>G (IVS5+10C>G) and the polymorphism c.472G>A (p.Glu158Lys). According to the literature [1-7] and the geneticist's report, this combination of genetic variants in heterozygosity could be linked to moderate or transient symptoms of trimethylaminuria. Biochemical urine analysis for TMA and TMAO was not available.

Following genetic confirmation, the parents were advised to reintroduce fish into the child’s diet in smaller portions and to adopt hygiene measures, including the use of low-pH soap, to help manage any potential odour. The complaints were successfully resolved with this strategy, and since he was 19 months old, he has been consuming the recommended portions of fish (five to six meals per week) without recurrence of the odour or any other symptoms. His growth and neurodevelopmental milestones continue to progress normally.

## Discussion

The present case illustrates a transient form of trimethylaminuria, likely resulting from a combination of heterozygous genetic variants in the FMO3 gene and the enzymatic immaturity characteristic of early childhood [1]. FMO3 activity gradually develops from birth, reaching full expression only around 11 years of age. This incomplete enzymatic maturation may explain why, in young children, exposure to a diet rich in TMA precursors can overwhelm hepatic oxidation capacity, resulting in transient episodes of the characteristic odour [1,2,6,7].

There is increasing evidence that trimethylaminuria may not be limited to a classic recessive disorder but rather represents a spectrum of phenotypes with variable expressivity [1,2,4]. The FMO3 gene is highly polymorphic, with genetic variations in both coding and non-coding regions that can influence enzyme functionality and contribute to the observed phenotypic spectrum in trimethylaminuria, with symptoms ranging from mild and transient to more severe forms [1,2,6]. In this patient, two genetic variants were identified: the common polymorphism p.Glu158Lys, associated with reduced enzyme activity, and an intronic variant, c.627+10C>G, whose functional significance remains uncertain. However, it cannot be excluded that variants in the intronic region may also contribute to interindividual differences in enzyme expression [1,2,5]. Although these polymorphisms do not typically cause disease per se, they can aggravate the phenotype, particularly in children, due to naturally low enzyme expression, which is likely the underlying cause in this case [1-3].

**Figure 1:**
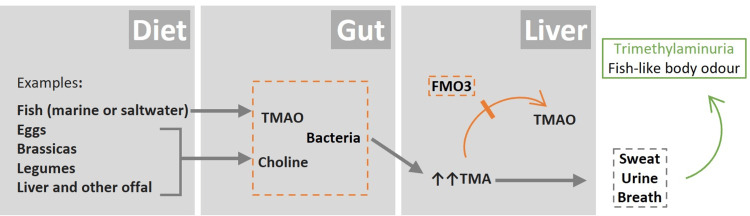
Primary metabolic pathway involved in trimethylaminuria. FMO3: flavin-containing monooxygenase 3; TMAO: trimethylamine-N-oxide; TMA: trimethylamine. Image credit: Dr. Resende. The structure of the schematic is based on Reference [2].

There is currently no cure for this disorder, and the primary therapeutic approach is dietary management, with specific restrictions on foods high in TMA precursors. Marine (sea or saltwater) fish, especially deep-sea fish with very high TMAO content, should be particularly avoided, along with foods high in choline, such as eggs, liver, kidney, peanuts, soy products, beans, peas, and other types of vegetables (Figure 1). In this case, the gradual reintroduction of fish into the diet in smaller portions allowed for the resumption of a normal diet without symptom recurrence, reflecting the effectiveness of dietary management in transient forms of the condition. It is also recommended to use low-pH (5.5-6.5) soaps and lotions to reduce skin odour. In cases with persistent symptoms or in circumstances where the production of TMA is increased, additional interventions, such as intermittent oral antibiotics (e.g., metronidazole or neomycin), may be indicated to modulate gut flora activity [5,7].

Although it is not associated with mortality or morbidity, the psychosocial consequences of this condition can be devastating, as it may severely impact the quality of life due to the associated odour and consequently lead to anxiety, depression, social isolation, and, in extreme cases, suicidal ideation [1,2,7].

Early diagnosis and intervention are critical for improving the quality of life of these patients. Genetic testing through molecular analysis is essential not only for confirming the diagnosis but also for understanding the mutational spectrum of the FMO3 gene and disease-associated phenotypes, which can guide healthcare professionals towards more individualised treatment strategies [1,2].

## Conclusions

Trimethylaminuria is a rare metabolic disorder that can significantly impact the quality of life due to its characteristic unpleasant odour. Raising awareness of this condition among healthcare professionals is crucial for ensuring prompt diagnosis and effective management. By implementing targeted dietary and hygiene strategies, patients can experience symptom relief, thereby reducing the associated psychosocial burden. Furthermore, the combination of genetic testing and urinary analysis of TMA and TMAO, when available, provides valuable insights into the condition, facilitating individualised and optimised patient care.
